# Predicting Protein Function in the AI and Big Data Era

**DOI:** 10.1021/acs.biochem.5c00186

**Published:** 2025-05-17

**Authors:** Riccardo Percudani, Carlo De Rito

**Affiliations:** † Department of Chemistry, Life Sciences and Environmental Sustainability, University of Parma, 43124 Parma, Italy

## Abstract

It is an exciting time for researchers working to link proteins to their functions. Most techniques for extracting functional information from genomic sequences were developed several years ago, with major progress driven by the availability of big data. Now, groundbreaking advances in deep-learning and AI-based methods have enriched protein databases with three-dimensional information and offer the potential to predict biochemical properties and biomolecular interactions, providing key functional insights. This progress is expected to increase the proportion of functionally bright proteins in databases and deepen our understanding of life at the molecular level.

## Graphical abstract

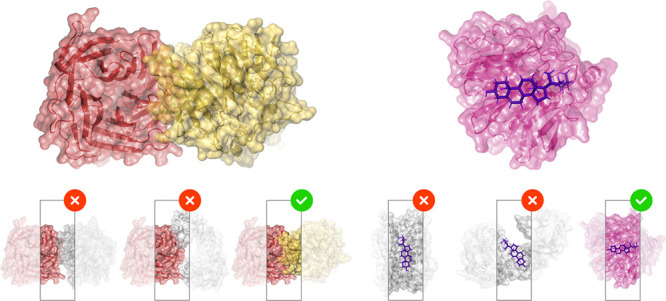

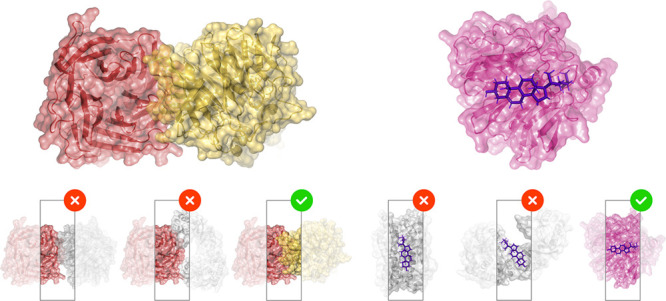

## Introduction

This perspective on recent advancements in protein function prediction is especially intended for experimental biologists. Particularly, those of you who are puzzled by a functionally uncharacterized protein pointed out by an experiment, searching for the protein corresponding to an unassigned density in a cryo-EM map, or trying to link a gene to a metabolic reaction or a rare disease. Some of you may be wondering: “*Shouldn’t I have more ways to find what I am looking for, now that a massive amount of digital information and AI tools are available?*”. The short answer is yes. The long one follows.

The challenge of linking proteins to their biological role arises in two ways: identifying the possible function of a known sequence or finding the sequence behind a known function.^1–3^ While the latter is facilitated by searching in a closed space—the information encoded in a genome—there are long-standing unsolved cases even in human phenotypes, including metabolic pathways^4,5^ and genetic diseases.^6,7^ Protein functional annotation,^8,9^ the first challenge, ranges from very easy to nearly impossible, mostly depending on information from homologous sequences. As a result, many database proteins remain functionally obscure.

Recent surveys indicate that only ∼20% of the nonredundant proteins in UniProt (UniRef50, which clusters 350 million sequences at 50% sequence identity) are functionally bright by virtue of having a characterized close homologue, while 34% are almost completely dark.^10^ The remaining proteins fall in between, with only partial information available. These figures improve slightly when considering well-studied organisms. For example, the Pharos database (https://pharos.nih.gov) classifies ∼5,500 human protein-coding genes (∼30%) as dark targets (TDark), although darkness in this case refers to the lack of dedicated studies.

Molecular biology entered the big data era at the start of the millennium with the advent of high-throughput genome sequencing.^11,12^ With a wide range of omics data now available, genomic data remain the most representative of the different life forms and are expected to maintain this central role in the future. The transition into the AI era is more recent, though early applications of machine learning paved the way.^13^ However, it is the advent of deep learning that has truly transformed the field, enabling the prediction of three-dimensional (3D) protein structures on a global scale. This breakthrough recognized last year by the Nobel Prize in Chemistry has added a new dimension to the genome information: 214 million 3D protein structures are currently available in the AlphaFold DB,^14^ and 772 million metagenomic structures are available at the ESM Atlas.^15^ In parallel, AI-based approaches are being applied for assigning protein names (https://www.uniprot.org/help/ProtNLM) and for predicting protein functions from sequence and structure.^16–19^

In this perspective, we will explore evidence for protein function that can be extracted from genome information and applied across the tree of life. Other types of big data—such as gene expression, protein localization, gene knockout (KO) phenotypes, whole-genome association studies, genome-phenome data, and medical records—offer powerful opportunities for inferring protein functions, though they are available for selected organisms.

## Function through Homology

Since its earliest applications, homology search has proven to be a most powerful tool for characterizing genes and proteins.^20^ Today, the vast majority of functional annotations in sequence databases rely on similarity to a small set of experimentally characterized molecules.^21^ This approach extends even to distant homologues, where the function of a human gene may be inferred, for example, from a bacterial gene that has been tested in the lab, honoring the Monod’s principle that “What is true for *Escherichia coli* is true for the elephant”.^22^ However, it is well-known that sequences can diverge to such an extent, even in related species, that homology becomes unrecognizable^23^ (Figure 1A).

**Figure 1:**
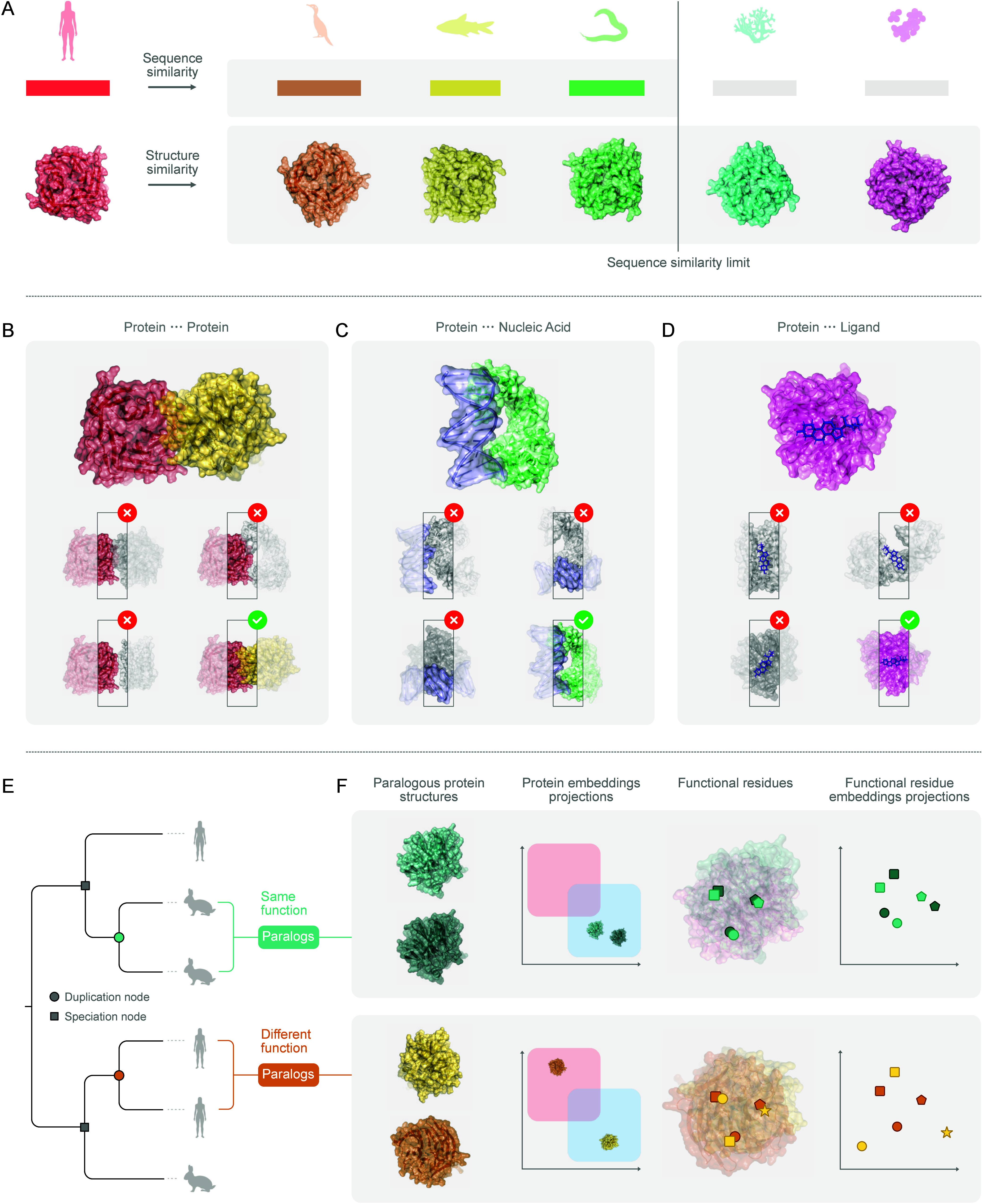
Novel opportunities in protein function prediction. **A**, Expanding homology. Sequence-based homology searches through pairwise comparisons are effective in identifying related sequences but can miss distant homologues due to divergence at the sequence level. Structure-based searches go deeper into the evolutionary relationships by leveraging 3D similarities. **B–D**, Biomolecular interaction screenings. Schematic examples of protein–protein (**B**), protein–nucleic acid (**C**), and protein–ligand complexes (**D**). Computational screenings can identify the correct partners by assessing confidence measures of biomolecular interactions. **E–F**, Functional shifts in protein evolution. Gene duplication generates paralogs that can either retain the same function or diverge (**E**). As illustrated by a simplified bidimensional projection, protein embedding vectors could reveal this shift (**F**), with functionally conserved paralogs clustering in the same functional space, while divergent ones occupying distinct regions. A more detailed analysis based on residue embedding pinpoint functional residues (shapes) showing conservation in isofunctional paralogs, and nonconservative substitutions in diverged ones.

The sensitivity limits of pairwise sequence comparisons can be overcome by methods that leverage multiple sequence alignments and statistical models of residue conservation, particularly Hidden Markov Models (HMMs).^24,25^ However, the structure of proteins is generally far more conserved than sequence throughout evolution. With the availability of protein structures at the genome scale and fast algorithms for structural comparisons,^26^ it is now possible to perform sensitive pairwise similarity searches at the 3D level (Figure 1A). This approach has been applied to the exploration of the natural protein universe and proved useful for the assignment of protein functions and the discovery of novel families and protein folds.^10^

Of course, identifying a homologous relationship—especially a distant one—is not sufficient to infer function. There are still many protein domains of unknown function (DUF), meaning no family member has been functionally characterized.^27^ Additionally, functional divergence is common in evolution, especially after gene duplication. Assigning function through homology often requires further evidence, such as a precise phylogenetic assessment (e.g., paralogy vs orthology) and a detailed analysis of functionally critical residues. The good news is that AI-based tools are increasingly providing valuable help in these analyses (see below).

## Function through Prediction of Macromolecular Complexes

As learned from structural genomics projects, the 3D structure of an uncharacterized protein only occasionally offers decisive insights into its function.^28,29^ By contrast, determining the structure of complexes often provides strong functional clues, linking previously uncharacterized proteins to known molecular processes. Recent examples are the identification through cryo-EM of TMEM106B as a component of amyloid filaments in the brain,^30^ and Tmem249 and Slco6c1 as components of the sperm cation channel complex.^31^ Because cryo-EM maps often derive from proteins extracted from natural sources, previously unknown components can be revealed along with their functions, also with the aid of dedicated software.^32,33^

Large-scale deep learning-based prediction of the structure of protein complexes was introduced with RoseTTAFold^34^ and further enhanced with AlphaFold multimer^35^ and AlphaFold 3 (AF3).^36^ Similar to cryo-EM, computational modeling of protein complexes can support functional inference, but at a broader scale. These tools enable high-resolution in silico screenings of protein–protein interactions (Figure 1B), with the potential to uncover new components of known complexes or entirely new assemblies. Such screenings have already been applied for instance to the identification of Yeast protein complexes,^37^ an essential factor for the assembly of the replication machinery,^38^ and components of the mRNA export pathway^39^ and the vertebrate fertilization complex.^40^

The last generation of deep learning models like AF3 now allow for the modeling of protein–nucleic acid complexes at scale, opening the door to in silico screenings of protein–DNA and protein-RNA interactions (Figure 1C). These approaches have the potential to complement experimental techniques such as CLIP-seq and ChIP-seq, facilitating the discovery of novel nucleic acid-binding proteins and the functional annotation of uncharacterized proteins. However, the accuracy of protein–nucleic acid predictions, particularly for RNA, remains lower than that for protein complexes,^36^ possibly due to the much lower number of experimentally solved structures available for model training.

In silico screening for macromolecular interactions can leverage both global (e.g., ipTM, interface predicted Template Modeling) and local (e.g., PAE, Predicted Aligned Error) confidence measures of the molecular interaction, and efficient algorithms for the combinatorial assembly of subunits.^41,42^ These screenings are computationally intensive and require high-end GPUs, which are costly and in high demand at HPC facilities. However, part of the computation, namely homologue and template searches, relies on CPUs, creating opportunities for pipeline optimization and hybrid resource usage.

The plethora of structural complexes expected to enrich biological databases in the coming years will entail comparing macromolecular assemblies across species. In anticipation of this, bioinformatics tools for large-scale comparisons of complexes have already been made available.^43^

## Function through Prediction of Protein–Ligand Interactions

Structure-based prediction of protein–ligand interactions represents another promising avenue for functional prediction, applicable across a broad range of protein classes, including receptors, transporters, and enzymes. Traditional approaches rely on molecular docking^44,45^ to predict potential ligands and their binding modes. Successful cases have been reported in which protein function was inferred by docking a set of ligands against a known protein structure,^46^ or conversely, by screening a set of protein structures against a known ligand.^47^

Possible limitations of docking-based techniques include the need of providing adequately prepared 3D structures (both for the ligand and the protein),^48^ and, in some cases, the definition of a region in the protein structure where the interaction with the ligand is expected to occur.^49,50^ AI-based tools promise to overcome many of these constraints and greatly facilitate the process by requiring just a protein sequence and the chemical structure of the ligand, usually provided in its SMILES (Simplified Molecular Input Line Entry System) form.

The ease of use of AI-based tools, combined with reported improvements in prediction accuracy,^36^ could boost large-scale screenings for discovering protein–ligand interactions (Figure 1D). Nevertheless, whether confidence measures of protein–ligand interactions have enough discriminatory power to distinguish among binding and not binding molecules in high-throughput settings is not well established. In addition, current AI implementations suffer some limitations “with respect to stereochemistry, hallucination, dynamics and accuracy for certain targets”.^36^ Stereochemistry violations include the tendency of producing clashing atoms and the possibility that ligand chirality is violated in the output model. Quite obviously, these limitations can compromise computational screenings as well as targeted prediction for certain ligands.

## Functional Shifts in Protein Evolution

Proteins that share a common ancestor—whether as orthologs via speciation or paralogs via gene duplication—can follow different evolutionary trajectories that either preserve ancestral functions or promote divergence.^51–53^ The evolutionary relationship between orthologs and paralogs can be visualized through phylogenetic trees; in some cases, proteins maintain their original biochemical functions, while in others, particularly after gene duplication, may acquire a new function (Figure 1E). These functional shifts are often caused by point mutations, domain rearrangements, and alterations in residue–residue interactions, all of which can be examined using comparative sequence and structure analyses.^54,55^

Detecting whether a protein has retained its original function or undergone divergence is a difficult task involving the analysis of the specific residues, motifs, or domain architectures responsible for activity. Traditional methods approach this problem through homology-based comparisons and the identification of conserved regions and residues. AI-driven strategies use advanced machine learning models and data integration to capture functional indicators.^16,17^ Protein large language models and learned embeddings^56–58^ can reveal patterns that traditional approaches might overlook, highlighting key residue positions implicated in functional shifts or maintenance. These models learn the “language” of proteins,^59^ generating both a global embedding that summarizes the entire protein sequence and residue-level embeddings that capture the contextualized properties of individual amino acids.

Protein embeddings are high-dimensional vectors representing sequences, generated by neural networks trained on large protein databases. These models, often based on transformer architectures, learn patterns of amino acid co-occurrence and context, capturing structural and functional features.^57^ When projected into embedding space, paralogs with conserved functions are expected to cluster together, while those with substantial functional divergence may occupy distinct regions (Figure 1F, left). At a more granular level, residue embeddings represent individual amino acids within their local sequence context, integrating chemical and physical properties such as hydrophobicity, charge, and polarity. These embeddings often encode information that correlates with functional relevance, enabling the identification of residues important for catalysis, structural stability, or interactions with other molecules.^60,61^ The residue embedding projections (Figure 1F, right) illustrates how those relevant residues cluster together in the case of conserved function and become more dispersed when functional divergence occurs.

## Guilty by Association

As uncovering connections between known offenders and their accomplices helps resolve collaborative crimes, detecting associations between different proteins can offer decisive clues about their functions. Biological processes rely on molecular collaboration within complexes or pathways and identifying such associations is key to elucidating protein roles.

Three foundational methods for detecting functional associations from genomic data have been proposed in the late 1990s: gene neighborhood,^62,63^ gene fusion,^64,65^ and co-occurrence (also known as gene coevolution or phylogenetic profiling).^65,66^ These pillars of functional inference correspond to the first three evidence channels (columns) of the STRING functional association table.^67^

Gene neighborhood infers functional relationships based on the genomic proximity of genes, particularly in prokaryotic genomes. In bacteria and archaea, genes involved in the same biological process or protein complex are often located near each other on the chromosome and cotranscribed as operons.^62,63^ When such arrangements are conserved across multiple species, it strongly suggests that the encoded proteins are functionally linked. While this type of analysis is generally not applied to eukaryotic genomes, the functional insights gained from prokaryotic gene neighborhoods can extend to eukaryotes when orthologous counterparts are present.^68^ Nevertheless, there are known examples of metabolic gene clusters in some eukaryotes,^69,70^ and even vertebrate genomes are rich in promoter-sharing head-to-head gene pairs, which are often an indication of functional coupling.^71^ Machine learning techniques have been shown to help functional inference based on gene neighborhood.^72^

Gene fusion, where two separate genes in one organism appear as a single, fused gene in others, can provide strong evidence of functional association. Despite the name, the origin of such arrangements may involve either fusion or fission events.^73^ This evidence applies both to eukaryotic and prokaryotic genomes, and functional inference generally requires that fused proteins are orthologs of the two nonfused ones. As illustrated by the remarkable example of animal fatty acid synthase, gene fusions are frequent in metabolic pathways,^74,75^ and can reveal enzymatic functions even when both fused partners are uncharacterized.^76^ A notable special case are somatic gene fusions in cancer, which can also offer valuable insights into protein function.^77,78^ A central tenet of evolutionary gene fusions is that the corresponding proteins are likely to physically interact also when encoded by separated genes.^64,65^ Interestingly, AI-based tools enable now to verify this assumption on a global scale.

Gene coevolution analysis, which identifies shared patterns of gene presence/absence across species, is likely the method that benefits most from the increasing size and taxonomic diversity of genomic data. However, its application presents several challenges, particularly in eukaryotes. Shared inheritance, for instance, produces false-positive associations where genes are shared due to vertical descent rather than functional linkage. At the other extreme, extensive gene loss, especially in parasitic or streamlined genomes introduces another confounding factor. Further complexity arises when functional links emerge or decouple in specific evolutionary branches, through processes such as gene neofunctionalization, moonlighting, or displacement, resulting in coevolutionary signals that can be masked in global comparisons.^79,80^ To address these challenges, gene coevolution methods are continuously being developed and refined.^81,82^ SVD-phy reduces noise and false positives by filtering less informative profiles^83^ (https://string-db.org/), the clade-wise approach detects local coevolution signals within specific eukaryotic lineages^79^ (https://tabachlab.shinyapps.io/CladeOScope/), and the cotransition (cotr) analysis focuses on correlated gene gains and losses, detecting sparse but statistically significant coevolutionary patterns.^80^ Transition-based approaches also reduce computational complexity, though efficient algorithms are available to speed up traditional computations.^84^ Finally, machine learning approaches to coevolutionary analysis have shown promise in outperforming traditional techniques.^85^ All these different approaches suggest that no single method can capture all coevolutionary signals. Using multiple methods can reveal associations that individual techniques might miss. A fundamental challenge in coevolutionary analysis is identifying the same gene across species, which requires distinguishing orthologs from paralogs—a task that may increasingly benefit from machine learning.^86,87^ Various orthology databases adopt different grouping strategies,^88–90^ which can lead to differences in downstream analyses.

A general note of caution for deductive inference based on gene/protein association methods is that, beyond the risk of false-positives, the very concept of “guilt by association” is a known fallacy in formal logic. Also in biological systems, associations may arise for various reasons, not necessarily related to the function being hypothesized.

## Some Current Limitations of Protein Databases

Although one is generally cautious as regard to functional annotation in protein databases, particularly for entries that have not undergone manual curation, the sequence itself is something in which one generally trusts. However, it is important to remember that the prediction of protein-coding genes in eukaryotic genomes is far from straightforward, due to the complexity of exon–intron structures and the presence of alternative splicing and alternative transcription start sites.^91,92^ To these complications, one may add the rare, but well documented occurrence of non-AUG start codons and selenocysteine-encoding UGA codons in eukaryotic genes.^93–96^

The use of deep learning methods has led to improvements in gene model prediction,^97^ although errors persist, also as a consequence of incomplete genome assemblies. Errors in protein coding sequences, such as missed proteins, incorrect gene boundaries, intron retention, exon skipping, and erroneously fragmented or fused of genes, are frequent in draft genomes also depending on the annotation methods,^98–100^ although even the human genome is not exempt.^101^ While inaccuracies affecting portions of the protein sequence may have a limited impact on homology detection, which typically relies on local sequence similarity, they can have a much greater impact on 3D structure prediction, and an even more pronounced effect on the prediction of protein–protein complexes.

Also other methods of functional inference are affected by protein sequence inaccuracies. Mistakenly fused proteins can provide a false gene fusion evidence, and false gene absence or presence can severely affect gene coevolution analysis. It has been reported that a substantial proportion of genes are falsely inferred to be absent, particularly in certain clades.^100^ Birds, for example, have GC-rich regions in their genome that are difficult to sequence and assemble, resulting in a large number of falsely absent genes.^102^ A less appreciated issue is the inclusion of pseudogenes in protein collections, especially those recently inactivated and carrying few mutations. RefSeq may override such defects assuming sequencing errors and tag the product as LOW QUALITY PROTEIN. A representative example is guinea pig L-gulonolactone oxidase (XP_012998768), which derives from a well-known pseudogene with a frameshift and two internal stop codons.^103^ In such cases, protein-to-genome alignment tools^104^ can recover the correct protein information directly from the genome.

These challenges highlight how crucial accurate protein information—both sequence and annotation—is for reliable functional predictions. Even minor errors can mislead researchers as well as computational tools involved in structure prediction, interaction modeling, and evolutionary analysis. Ensuring high-quality database information is therefore essential not only to prevent mistakes, but also to support the discovery of novel biological functions.

## Some Current Limitations of AI-Based Functional Annotation

While AI-based protein function prediction tools have greatly improved in propagating known functions, they still face major challenges in automatically predicting truly novel functions. Most current models rely heavily on patterns learned from existing annotations, which limits their ability to generalize beyond known biochemical space. Therefore, the prediction of entirely new functions based on bioinformatics evidence still largely depends on the intuition and educated guesses of expert researchers, followed by experimental validation. However, as models become better at integrating structural, evolutionary, and biochemical data, there is growing potential for AI to contribute meaningfully to the discovery of entirely new functions in the future.
